# γH2Ax Expression as a Potential Biomarker Differentiating between Low and High Grade Cervical Squamous Intraepithelial Lesions (SIL) and High Risk HPV Related SIL

**DOI:** 10.1371/journal.pone.0170626

**Published:** 2017-01-24

**Authors:** Konstantinos Leventakos, Sotirios Tsiodras, Theodore Kelesidis, Maria Kefala, Christine Kottaridi, Aris Spathis, Alina-Roxani Gouloumi, Abraham Pouliakis, Asimakis Pappas, Vasileios Sioulas, Charalambos Chrelias, Petros Karakitsos, Ioannis Panayiotides

**Affiliations:** 1 2^nd^ Department of Pathology, University Hospital “Attikon”, School of Medicine, National and Kapodistrian University of Athens, Athens, Greece; 2 4^th^ Department of Internal Medicine, University Hospital “Attikon”, School of Medicine, National and Kapodistrian University of Athens, Athens, Greece; 3 Department of Cytopathology, University Hospital “Attikon”, School of Medicine, National and Kapodistrian University of Athens, Athens, Greece; 4 3^rd^ Department of Obstetrics and Gynecology, University Hospital “Attikon”, School of Medicine, National and Kapodistrian University of Athens, Athens, Greece; Universidade Estadual de Maringa, BRAZIL

## Abstract

**Background:**

γH2AX is a protein biomarker for double-stranded DNA breakage; its expression was studied in cervical squamous intraepithelial lesions and carcinomas.

**Methods:**

Immunostaining for phospho-γH2AX was performed in sections from histologically confirmed cervical SIL and carcinomas, as well as from normal cervices used as controls. In total, 275 cases were included in the study: 112 low grade SIL (LGSIL), 99 high grade SIL (HGSIL), 24 squamous cell carcinoma (SCC), 12 adenocarcinoma and 28 cervical specimens with no essential lesions. Correlation of histological grading, high risk vs. low risk HPV virus presence, activated vs. non-activated status (by high risk HPV mRNA expression) and γH2AX expression in both basal and surface segments of the squamous epithelium was performed.

**Results:**

Gradual increase of both basal and surface γH2AX expression was noted up from normal cervices to LGSIL harboring a low risk HPV type, to LGSIL harboring a high risk virus at a non-activated state (p<0.05). Thereafter, both basal and surface γH2AX expression dropped in LGSIL harboring a high risk virus at an activated state and in HGSIL.

**Conclusions:**

γH2AX could serve as a potential biomarker discriminating between LGSIL and HGSIL, as well as between LGSIL harboring high risk HPV at an activated state.

## Introduction

Infection by HPV is responsible for the development of cervical squamous intraepithelial lesions (SIL) and cervical carcinoma, as well as for other squamous cell carcinomas in the anogenital and the head and neck area. There is an ongoing quest for biomarkers predicting the eventual progression of HPV related SIL into carcinoma.

Dysregulation of the DNA damage response (DDR) mechanism has been implicated in the pathogenesis of a multitude of malignancies and is currently investigated, in order to identify predictors of disease progression as well as novel therapeutic targets [1].

Genomic instability is important in the pathogenesis of cervical cancer and it is driven by the integration of DNA from high risk HPV types [1]. The disruption of post-transcriptional regulation of cyclin dependent kinase inhibitors leading to cell senescence and transformation, as well as inactivation of the major tumor suppressor gene p53 are among the crucial events of carcinogenesis [2]. At the molecular level, genomic instability has been associated with the formation of DNA double-strand breaks (DSBs) [1]; the DDR pathway exerts anti-tumor action by inducing p53-dependent cell cycle arrest, apoptosis and senescence [1]. The activated protein kinase ATM and its downstream target proteins including p53, the histone H2aX and checkpoint kinases play an important role in the DDR process by eliminating cells with damaged DNA early in the carcinogenesis process [1].

Early protein markers of tumorigenesis and their expression in HPV associated premalignant lesions may lead to better understanding of the pathogenesis of malignancy as well as better and earlier therapeutic interventions. Recently, several markers—including p53, Ki67 and γH2AX among others—have been used to this effect in HPV related tumorigenesis [3].

Among the key proteins in DNA repair, γH2AX which is the phosphorylated form of the histone protein H2Ax exerts its actions at nascent DSB sites; there, large numbers of the phosphorylated molecule create a focus around the DNA break sites, where accumulation of DNA repair and chromatin remodeling proteins occurs [4]. Thus, γH2AX has been used as a well-recognized protein biomarker for double-stranded DNA breakage [5]. Due to scarce data concerning γH2AX expression status in cervical SIL and carcinoma, the objective of this study was to investigate this expression, as well as its relation to the HPV type and HPV mRNA presence.

## Methods

We examined histologically confirmed cervical squamous intraepithelial lesions (SIL), squamous cell carcinomas (SCC) and adenocarcinomas (AdC) available through a prospective HPV registry operating in the “Attikon” University Hospital and maintained by the Departments of Cytopathology and Pathology of the hospital [6]. Moreover, cases within normal limits were included for comparison purposes. Specifically, control and SIL cases concerned specimens received in a 3- year period, i.e. from January 2010 up until December 2012 included; SCC and AdC samples were retrieved from the totality of the files of the Department of Pathology, i.e. from July 2003 up to December 2015 included. The “Attikon” University Hospital Institutional Ethics Review Board approved the study, in compliance with the Helsinki declaration. Written informed consent was obtained from all participating patients.

For cytology analysis, liquid based cytology (LBC) samples were processed with the ThinPrep® method as previously described [6].

HPV DNA detection was performed in ThinPrep® samples as previously described [7]. Briefly a commercially available kit that identifies and types the 35 most common HPV types (6, 11, 16, 18, 26, 31, 33, 35, 39, 40, 42, 43, 44, 45, 51, 52, 53, 54, 56, 58, 59, 61, 62, 66, 68, 70, 71, 72, 73, 81, 82, 83, 84, 85 and 89) was used (CLART2 HPV, Genomica, Spain).

HPV mRNA detection was performed in ThinPrep® samples using a commercially available kit (HPV OncoTect™ E6, E7 mRNA Kit, In Cell Dx, U.S.A.) that utilizes in-situ hybridization for the identification of HPV mRNA of the two oncogenes [8] as previously described [9]. In negative or inadequate samples, a second mRNA detection technique, that uses nucleic acid sequence based amplification (NASBA, NucliSENS Easy Q HPV, Biomerieux, France) was used as previously described [10]. If either of the two mRNA tests were positive, the sample was considered as positive.

Biopsies were obtained by expert gynecologists during colposcopy procedures, as well as from surgical specimens through either conization or hysterectomy. All women with indications consented to this procedure.

Specimens were fixed in a 10% buffered formol solution. All cases were diagnosed by the same qualified (more than 20 years of experience) pathologist, according to the two-tiered Bethesda terminology, in use as of January 2010, for histology reports concerning SILs, in accordance with the relevant AFIP fascicle; the superiority of this system over the previous, three-tiered cervical intraepithelial neoplasia (CIN) has been repeatedly proven with a recent report depicting an inter-observer variability (kappa index) of 0.68 versus 0.46 [11,12]. Although performed, immunostaining both for Ki67 and p16 were used only as adjunctive, non-confirmatory parameters, given the controversy concerning interpretation of p16 immunostaining in histological sections [13].

Four μm-thick deparaffinized sections were stained with Hematoxylin and Eosin and immunostained with antibodies against phospho-γH2AX (mouse monoclonal. clone 9F3, ABCAM, 1:2500 dilution) and Ki67 (mouse monoclonal, clone MIB-1, DAKO, 1:100 dilution);.

For γH2AX, slides were immersed in a high pH target retrieval solution [K8004 [DAKO]), boiled in the PTLink (DAKO) at 97^°^C for 20 min and subsequently allowed to cool at room temperature for 20 min, following which immunostaining was performed in the DAKO Autostainer Link 48 using the EnVisionTM detection and visualization kit (K5007 [DAKO]).

For Ki67, antigen retrieval pretreatment with BondTM Epitope Retrieval Solution 2 was performed (pH 8.9 at 25oC), following which immunostaining was performed in the automated Leica Bond-Max system.

A light Hematoxylin counterstain was performed for both immunostains.

All immunostained slides were simultaneously assessed by two trained (3 years experience) pathologists with comparable experience (MK, ARG), as well as the above mentioned qualified pathologist (IP). For controls and SIL cases, lower (basal) and higher (surface) segments of the squamous epithelium were independently assessed; immunopositive cells had only nuclear staining. In every case, the percentage of immunopositive nuclei in 100 consecutive cells was calculated, both for the lower and higher segments of the epithelium. Since discrimination between lower and higher epithelial segments was not possible in carcinoma cases, the percentage of positive nuclei was calculated for a hundred consecutive cells.

We analyzed cytology data separately and dichotomized it into cytological high grade (i.e. ASC-H, HGSIL, SCC, AdC) and low grade lesions; we then correlated cytological with histological findings. Histological grading was dichotomized for comparison between low grade and high grade squamous intraepithelial lesions ((LGSIL and HGSIL respectively). Demographic parameters between patients according to histological grading and type of virus were performed by analysis of variance (ANOVA) and Kruskal-Wallis or chi-square test as appropriate. Further, patients were characterized as LGSIL harboring high-risk HPV types (LGSIL-HR) vs LGSIL harboring low risk virus (LGSIL-LR) and by whether they had activated or inactivated infection by means of mRNA positivity for one of the two oncoproteins. Correlation between histological grading, high risk vs. low risk virus presence, activated vs. non-activated status and γH2AX expression was performed using chi-square test and ANOVA as appropriate. In order to find whether a statistically significant difference existed in the γH2AX expression between the lower and higher epithelial segments of SILs, independent samples t-testing was performed. SPSS version 22.0 for Windows software (IBM Corp. Released 2013. IBM SPSS Statistics for Windows, Version 22.0. Armonk, NY: IBM Corp.) was used for data analysis. All statistical tests were two-tailed.

## Results

In total, 275 cases (cervical biopsies or hysterectomy specimens) were included in the study: 112 LGSIL, 99 HGSIL, 24 SCC, 12 AdC and 28 cervical specimens with no essential lesions (S1 Dataset). The mean age of the 275 subjects studied was 38.7±13.3 years (range 18–81 years). Table 1 depicts the association between cytological and histological diagnosis in the study sample. Overall, cytology results correlated well with histology (Table 1). For example, cytological high grade lesions (i.e. ASC-H, HGSIL, SCC, AdC) correlated strongly with histological high grade lesions (OR: 28; 95% CI 13.5–58.6, p<0.001). High risk HPV types were mostly noted in high grade histological lesions. HPV-16 was noted in 35 out 99 (35.4%) in samples from histologically confirmed HGSIL cases vs. 29/112 (25.9%) in histologically confirmed LGSIL cases, p<0.001]. On the other hand, low risk types predominated in histologically confirmed low grade lesions (p = 0.01, data not shown). Mixed infections with multiple HPV types were identified from both low grade [60/112, (53.6%)] and high grade lesions [35/99 (35.4%)].

**Table 1:** Cytological vs. histological classification for the studied samples. Rates are given in percentage (%) for the histological category.

	Cytological category								
Histological category	WNL	ASCUS	LGSIL	ASC-H	HGSIL	SCC	AdenoCa	Not available	Total
NEGATIVE	10 (35.7%)	9 (32.1%)	5 (17.9%)	2 (7.1%)	2 (7.1%)	0 (0%)	0 (0%)	0 (0%)	28 (10.2%)
LGSIL	17 (15.2%)	24 (21.4%)	62 (55.4%)	2 (1.8%)	7 (6.3%)	0 (0%)	0 (0%)	0 (0%)	112 (40.7%)
HGSIL	3 (3%)	2 (2%)	19 (19.2%)	7 (7.1%)	62 (62.6%)	0 (0%)	0 (0%)	6 (6.1%)	99 (36%)
SCC	0 (0%)	0 (0%)	0 (0%)	0 (0%)	3 (12.5%)	9 (37.5%)	1 (4.2%)	11 (45.8%)	24 (8.7%)
ADENO-CA	0 (0%)	1 (8.3%)	0 (0%)	0 (0%)	1 (8.3%)	0 (0%)	7 (58.3%)	3 (25%)	12 (4.4%)
**Total**	**30 (10.9%)**	**36 (13.1%)**	**86 (31.3%)**	**11 (4%)**	**75 (27.3%)**	**9 (3.27%)**	**8 (2.91%)**	**20 (7.27%)**	**275 (100%)**

**Fig 1:**
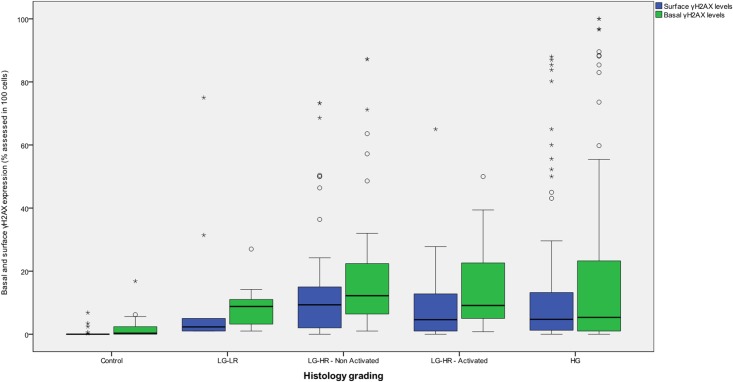
Basal and surface γH2AX expression (% positivity in 100 assessed cells) according to histological grade.

**Fig 2:**
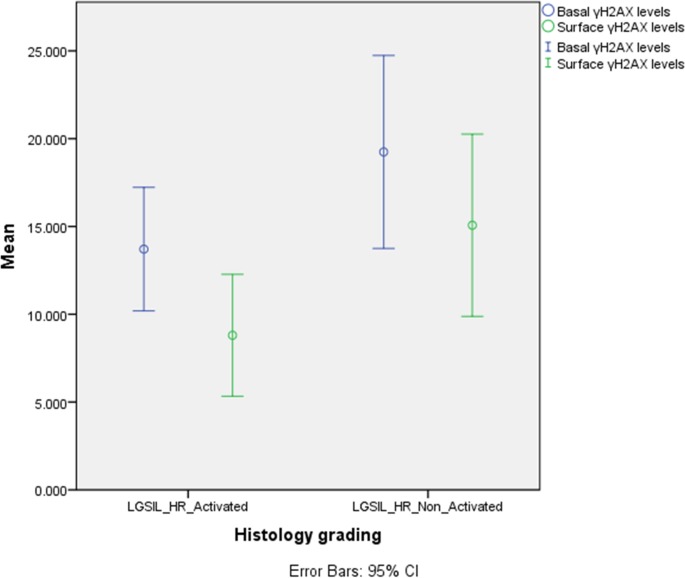
Mean value and 95% confidence intervals, of γH2AX expression for the basal and surface components for histological LGSIL cases with HR HPV infection, activated (N = 51) and non-activated cases (N = 57).

**Fig 3:**
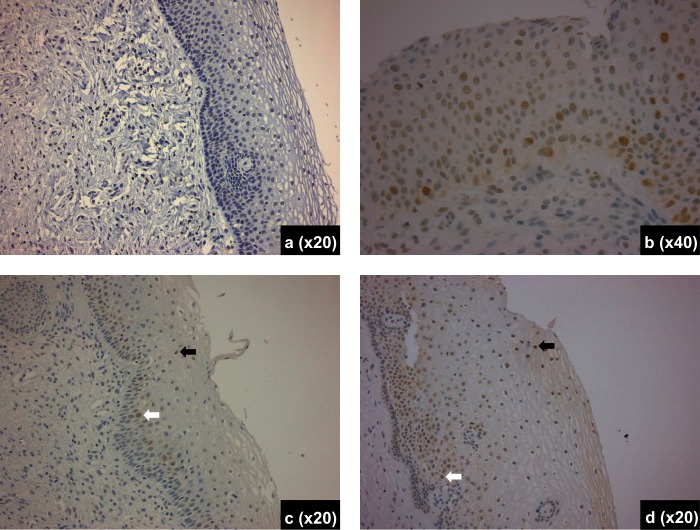
γ2ΗΑΧ immunostaining of histological sections from the following cases: control (a); HGSIL (b); LGSIL harboring High Risk HPV at an activated state (c); and LGSIL harboring High Risk HPV at a non activated state (d). In pictures c and d, arrows depict positive cell (nuclear staining) at basal (white arrow) and surface (black arrow) area of the epithelium. Objective magnifications appear on pictures.

Lesions were then categorized as harboring viruses at an activated vs. non activated status, depending on mRNA positivity. Out of 108 negative /inadequate samples with Oncotect, 23 (21.3%) tested positive with NucliSens. Differences were then sought in LGSIL harboring a high-risk HPV type, depending on whether they were identified as non-activated (n = 56) or activated (n = 46). (Tables 2 and 3). Table 2 depicts γH2AX expression (basal and surface) for the different types of histological lesions. Sections from cervices with no essential lesions had significantly less basal and surface γH2AX expression compared to LGSIL harboring a high risk virus (LGSIL-HR) and HGSIL (Table 2 and Figs 1–3).

**Table 2:** γH2AX expression (basal and surface segments of epithelium) across histologically confirmed lesions for all patients with available data. Statistically significant associations (p<0.05) by ANOVA are flagged.

Histological grading	Age in yrs, median (IQR)	γH2AX Basal, median (IQR)	γH2AX Surface, median (IQR)
***Normal*, *n = 28 (10*.*2)***	45 (30.3–48.8)	0.3 (0–2.6)^&^^,^^^^^,^^%^	0 (0–0)^$^^,^ ^&^
*LGSIL*, *n = 112 (40*.*5%)*	33 (26–41)	10.5 (5.4–22)	6.3 (1–15)
***LGSIL-LR*, *n = 10/112 (8*.*9%)***	31 (27–43.8)	8.8 (3–11.8) ^%^	2.3 (1–11.6)
*LGSIL-HR*, *n = 102/112 (91*.*1%)*	33 (26–41)	11 (5.6–22.6)*^,^ ^%^	7.5 (1–15)*
***LGSIL-HR*, *non activated*, *n = 56/102 (54*.*9%)***	32.5 (25–40)	12.2 (6.4–22.8)	9.3 (1.9–15.7)
***LGSIL-HR*, *activated*, *n = 46/102 (45*.*1%)***	33 (26–42)	9.1 (5–23.1)	4.6 (1–12.9)
***HGSIL*, *n = 99 (36*.*1%)***	34 (28–42)	5.3 (1–23.5)*	4.7 (1.2–13.4)*
***SCC*, *n = 24 (8*.*8%)***	56.5 (46.3–67)	13 (5.8–24.6)*	N/A
***AdC*, *n = 12 (4*.*4%)***	64.5 (56–72.5)	15.6 (6.1–30.8)*^,^ ^#^^,^ ^$^	N/A

LGSIL: Low grade Squamous Intraepithelial Lesion; HGSIL: High grade Squamous Intraepithelial Lesion; SCC: Squamous cell carcinoma; AdC: Adenocarcinoma

* For comparison between Normal and flagged histological lesion;

# For comparison between LGSIL-LR and flagged histological lesion;

$ For comparison between LGSIL-HR and flagged histological lesion;

& For comparison between HGSIL and flagged histological lesion;

^ For comparison between SCC and flagged histological lesion;

% For comparison between AdC and flagged histological lesion

**Table 3:** Histologically confirmed low grade cases (LGSIL) where high risk (HR) HPV type infection (LGSIL-HR) was identified. Comparison between activated (mRNA positive) and non-activated cases (mRNA negative) for 102 subjects. Statistically significant association are flagged with an asterisk.

Population characteristics	LGSIL-HR Non Activated	LGSIL-HR Activated	p
N = 102, n (%)	56 (54.9)	46 (45.1)	
Age, median (IQR)	32.5 (25–40)	33 (26–42)	0.9
γH2AX Basal, n = 102, median value (IQR)	12.2 (6.4–22.8)	9.1 (5–23.1)	0.099
γH2AX Superficial, n = 102, median value (IQR)	9.3 (1.9–15.7)	4.6 (1–12.9)	0.03*
HPV-16, n = 29, n (%)	10 (34.5)	19 (65.5)	0.01 *
HPV-18, n = 16, n (%)	10 (62.5)	6 (37.5)	0.6
HPV-31, n = 9, n (%)	6 (66.7)	3 (33.3)	0.5
HPV-33, n = 6, n (%)	2 (33.3)	4 (66.7)	0.4
HPV-45, n = 7, n (%)	3 (42.9)	4 (57.1)	0.7
*HPV-6*, n = 3, n (%)	2 (66.7)	1 (33.3)	1
*HPV-11*, n = 2, n (%)	2 (100)	0 (0)	0.5
HPV Other HR (not HPV-16, HPV-18, HPV-31, HPV-33 or HPV-45), n = 74, n (%)	45 (60.8)	29 (39.2)	0.07
HPV mixed (has multiple infections irrelevant of HR or LR), n = 58, n (%)	30 (51.7)	28 (48.3)	0.5

IQR: interquartile range

Gradual increase of both basal and surface γH2AX expression was noted up from LGSIL harboring a low risk HPV type (LGSIL-LR) to LGSIL harboring a high risk virus at a non-activated state (LGSIL-HR- non Activated, Figs 1–3).

Thereafter, both basal and surface γH2AX expression dropped in LGSIL harboring a high risk virus at an activated state (LGSIL-HR-Activated) and in HGSIL (Fig 1). There was a higher surface γH2AX expression in LGSIL cases with HR HPV infection at a non—activated state (as identified by the mRNA expression) versus LGSIL cases with HR HPV infection at an activated state (p = 0.03, Figs 2 and 3). Variability in the difference in expression was noted across individual HPV types (Table 3); however, this difference remained statistically significant (i.e. increased protein expression in LGSIL cases with HR HPV infection at a non—activated state) when examining the most important high-risk HPV types as a group i.e. HPV-16, 18, 31, 33, and 45 together (data not shown).

Concerning carcinomas γH2AX expression was significantly higher when compared with most of the other histological lesions (Table 2).

The distribution of HPV types in LGSIL according to HPV type and mRNA positivity is shown in Table 3. HPV-16 was more prevalent in LGSIL harboring a high risk type with mRNA positivity (OR 3.3, 95% CI 1.3–7.9, p = 0.01, Table 3).

## Discussion

We assessed the expression of the histone 2AX phosphorylated at serine-139 (γH2AX) protein in cervical histological lesions of increasing grade. We found a gradual increase of both basal and surface γH2AX expression up from normal cervices to LGSIL harboring a low risk HPV type, to LGSIL harboring a high risk virus at a non-activated state. Thereafter, both basal and surface γH2AX expression dropped in LGSIL harboring a high risk virus at an activated state and in HGSIL. Moreover, there was a higher surface γH2AX expression in LGSIL cases with HR HPV infection at a non—activated state (as identified by the mRNA expression) versus LGSIL cases with HR HPV infection at an activated state.

Complex chromosomal changes and DNA damage are observed in high-risk HPV-associated malignancies [14]. Endogenous DNA damage can be caused by various stressors [15] and aberrant expression of DNA repair enzymes can cause persistence of damaged DNA and oncogenesis [15]. The regulation of the viral late phase that depends on the differentiation of the epithelial cell of the host, is provided by maintaining the competence of the cell cycle in suprabasal cells and the activation of the Ataxia-Telangiectasia Mutated (ATM) DNA damage response [16]. In contrast to migrating from the basal layer normal cells, that exit the cell cycle upon differentiation, HPV infected basal cells remain active and re-enter the S/G2 phase in suprabasal layers to replicate both viral and cellular genomes in a process called amplification [16].

**Fig 4:**
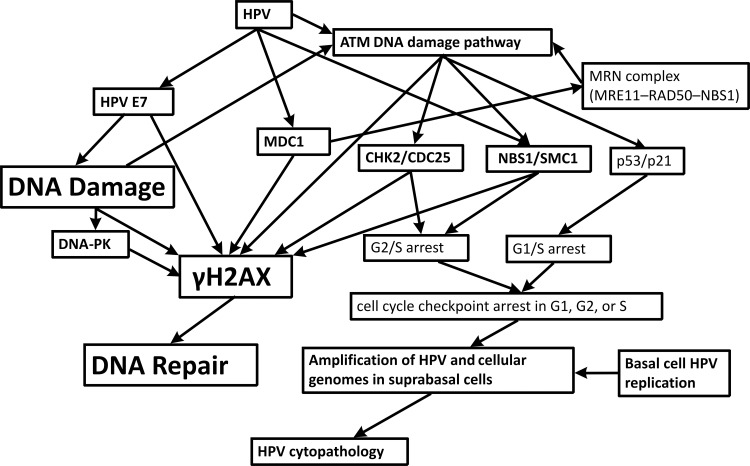
γH2AX as a regulator of cellular responses to HPV infection. An ATM-dependent DNA damage response is induced by HPV; however it is unclear which set of the ATM factors provide necessary functions. Complex feedback loops induce the phosphorylation of H2AX, that is recruited to regions flanking sites of double strand breaks. Proteins known to directly bind to / interact with H2AX are marked in bold. γH2AX forms complexes with these proteins associated with the viral DNA in HPV positive cells. Many of these loops interact with the HPV genome amplification that occurs during cycle arrest in late S/G2.

We attempted to evaluate DNA damage by investigating the presence of cells with nuclei exhibiting positive immunostaining for γH2AX. The histone variant H2AX is an important factor in preserving genome integrity in the mammalian genome [17]. Phosphorylation of γH2AX is an acute physiologic response to double strand breaks (DSBs) of the DNA leading to structural alterations at the damaged site; DNA repair proteins are recruited to these sites [17]. γH2AX has a major role in cellular responses to HPV infection that originate from an ATM-dependent DNA damage response pathway that is important for the amplification of the virus in differentiating cells [18]. Several *in vitro* and *in vivo* [19] studies have established the role of complex feedback loops (summarized in Fig 4) involving E7 HPV oncoproteins [14] and cellular proteins involved in the DNA repair pathways such as the ATM pathway [20,21], CHK2/CDC25 and NBS1/SMC1 [20], p53/p21[22], DNA-PK[23], MDC1[24], the MRN complex (MRE11–RAD50–NBS1) that directly and/or indirectly interact with γH2AX [24,25]. The generation of a positive feedback loop from these processes drives further phosphorylation of H2AX away from the DNA damage site.

It has been suggested that H2AX has both structural and functional roles in chromatin de-condensation and the repair process nearby the DSB [26,27]. Its absence has been associated with genomic instability as well as rapid oncogenicity (in conjuction with p53 deficiency) in animal experiments [28] [29]. Nevertheless, recent data suggest that the histone variant H2AX has additional roles in numerous biological processes beyond the canonical DNA DSB response, including some of the phases of cell division, the biology of the stem cell and aging [30]. It is likely that induced or naturally occurring DSBs act as the inciting event in H2AX phosphorylation; afterwards H2AX may affect other biological functions not always related to the DNA DSB response [30]. The type of biological function promoted depends on the specific cell type, the context within chromatin remodeling occurs as well as the presence of other remodeling complexes [30]. Thus, the role of γH2AX as a biomarker for certain cellular responses (including response to HPV) and its effects on biologic functions need to be further elucidated.

Histone phosphorylation produces foci that can be microscopically observed after specific antibody labeling [31,32]. This ability to use γH2AX foci to locate a DSB has enabled a better evaluation of DNA damage (even at low levels) along with repair and response mechanisms in an effort to develop novel predictive assays for the response to cellular injury.

Based on the above data, we attempted to evaluate the presence of DNA damage by quantifying the percentage of γH2AX immunopositive cells, as a surrogate marker for DNA DSBs, as well as to determine whether γH2AX can be used as a biomarker to identify early HPV lesions with high-risk oncogenic potential.

Our data suggest that there is a different expression of the γH2AX between the basal and surface components in SILs of increasing severity. After an initial increase, potentially corresponding to a γH2AX response to inciting cellular events, a decreased expression occurs in LGSIL harboring a high risk HPV type (LGSIL-HR) at an activated state. The observed decrease of γH2AX expression between LGSIL and HGSIL could be attributed to a significantly advanced extent of DNA damage in HGSIL compared to LGSIL, possibly no longer amenable to repair mechanisms, hence a trend towards a lower expression of this protein; the expression range across HGSIL lesions could be attributed both to the extent of the lesion (most biopsies harboring limited areas of HGSIL) as well as to other factors promoting H2AX phosphorylation (vide infra). The same hypothesis could also account for the lower expression of γH2AX in LGSIL cases associated with an activated HPV (LGSIL-HR activated) compared to those associated with a non-activated state (LGSIL-HR non-activated).

Although an increased expression of γH2AX was noted in in squamous cell carcinomas and adenocarcinomas compared to LGSIL we cannot use this finding for differentiation between them since there is a significant difference in structure between these lesions. More specifically, whereas in LGSIL, which is an intraepithelial lesion, it is possible to delineate a basal and a superficial half of the epithelium, this is no longer the case for SCC or AdC due to the disordered invasive growth. Therefore, a comparison of γΗ2ΑΧ in basal and superficial parts of the epithelium between various intraepithelial lesions, such as the one we performed in our work, is no longer possible for carcinomas. The fact that the protein expression was significantly higher in carcinomas comparing to other histological lesions reflects, in all probability, the multiple DNA damage events involved in this advanced stage of carcinogenesis.

Our study is limited by the small number of observations for validating a novel biomarker. Due to the lack of pilot data, power calculations could not be performed. Therefore, we chose to include consecutive samples received over a defined 3 year period, apart from carcinomas, where due to their rarity, all relevant samples from the archives of the Department of Pathology were retrieved. Data presented can be used for power calculations in future studies. Nevertheless, the inclusion of a high number of HGSIL, as well as the combination of two methods ensuring both broad range and high sensitivity of mRNA overexpression, adds power to this study. Furthermore, our results were corroborated when examining lesions associated with the most important high-risk HPV types, such as *HPV-16*, *18*, *31*, *33* and *HPV-45* as a group. Nevertheless, future studies will be necessary for further verification of these findings and evaluation of potential concomitant confounders.

We believe that the findings of our study are secondary to HPV infection and not due to differences in apoptosis or other regulators of γH2AX. Although γH2AX has been also reported to be induced during apoptotic DNA fragmentation, histological signs of apoptosis, such as nuclear condensation and fragmentation, were not seen in γH2AX positive foci, their absence suggesting that these foci represent cells that have acquired DNA damage.

In conclusion, understanding of the mechanism by which repair enzymes contribute to HPV genome amplification may provide further insight in the HPV related DNA damage response that may be involved in the development of malignancy [33]. Overall, our study identifies γH2AX as a potential diagnostic marker discriminating between LGSIL and HGSIL, as well as for the identification of LGSIL harboring high risk HPV at an activated state. Verification of this data in larger population samples may contribute to the evidence-based use of this biomarker in daily clinical practice.

## Supporting Information

S1 Dataset The minimum dataset of the current study. (SAV)

## References

[1] Curtin NJ (2012) DNA repair dysregulation from cancer driver to therapeutic target. Nat Rev Cancer 12: 801–817. 10.1038/nrc3399 23175119

[2] Yugawa T, Kiyono T (2009) Molecular mechanisms of cervical carcinogenesis by high-risk human papillomaviruses: novel functions of E6 and E7 oncoproteins. Rev Med Virol 19: 97–113. 10.1002/rmv.605 19156753

[3] Carlson BC, Hofer MD, Ballek N, Yang XJ, Meeks JJ, et al (2013) Protein markers of malignant potential in penile and vulvar lichen sclerosus. J Urol 190: 399–406. 10.1016/j.juro.2013.01.102 23399649

[4] Bonner WM, Redon CE, Dickey JS, Nakamura AJ, Sedelnikova OA, et al (2008) GammaH2AX and cancer. Nat Rev Cancer 8: 957–967. 10.1038/nrc2523 19005492 PMC3094856

[5] Bonner M, Strouse B, Applegate M, Livingston P, Kmiec EB (2012) DNA damage response pathway and replication fork stress during oligonucleotide directed gene editing. Mol Ther Nucleic Acids 1: e18 10.1038/mtna.2012.9 23343929 PMC3381643

[6] Bountris P, Haritou M, Pouliakis A, Margari N, Kyrgiou M, et al (2014) An intelligent clinical decision support system for patient-specific predictions to improve cervical intraepithelial neoplasia detection. Biomed Res Int 2014: 341483 10.1155/2014/341483 24812614 PMC4000928

[7] Tsiodras S, Hatzakis A, Spathis A, Margari N, Meristoudis C, et al (2011) Molecular epidemiology of HPV infection using a clinical array methodology in 2952 women in Greece. Clin Microbiol Infect 17: 1185–1188. 10.1111/j.1469-0691.2011.03505.x 21595788

[8] Narimatsu R, Patterson BK (2005) High-throughput cervical cancer screening using intracellular human papillomavirus E6 and E7 mRNA quantification by flow cytometry. Am J Clin Pathol 123: 716–723. 15981811

[9] Spathis A, Kottaridi C, Chranioti A, Meristoudis C, Chrelias C, et al (2012) mRNA and DNA detection of human papillomaviruses in women of all ages attending two colposcopy clinics. PLoS One 7: e49205 10.1371/journal.pone.0049205 23166611 PMC3499555

[10] Koliopoulos G, Chrelias C, Pappas A, Makridima S, Kountouris E, et al (2012) The diagnostic accuracy of two methods for E6&7 mRNA detection in women with minor cytological abnormalities. Acta Obstet Gynecol Scand 91: 794–801. 10.1111/j.1600-0412.2012.01414.x 22486415

[11] Stripp BR (2008) Hierarchical organization of lung progenitor cells: is there an adult lung tissue stem cell? Proc Am Thorac Soc 5: 695–698. 10.1513/pats.200801-011AW 18684719 PMC2645261

[12] Voltaggio L, Cimino-Mathews A, Bishop JA, Argani P, Cuda JD, et al (2016) Current concepts in the diagnosis and pathobiology of intraepithelial neoplasia: A review by organ system. CA Cancer J Clin 66: 408–436. 10.3322/caac.21350 27270763

[13] Clark JL, Lu D, Kalir T, Liu Y (2016) Overdiagnosis of HSIL on cervical biopsy: errors in p16 immunohistochemistry implementation. Hum Pathol 55: 51–56. 10.1016/j.humpath.2016.04.010 27134110

[14] Duensing S, Munger K (2002) The human papillomavirus type 16 E6 and E7 oncoproteins independently induce numerical and structural chromosome instability. Cancer Res 62: 7075–7082. 12460929

[15] Hoeijmakers JH (2001) Genome maintenance mechanisms for preventing cancer. Nature 411: 366–374. 10.1038/35077232 11357144

[16] Sakakibara N, Chen D, McBride AA (2013) Papillomaviruses use recombination-dependent replication to vegetatively amplify their genomes in differentiated cells. PLoS Pathog 9: e1003321 10.1371/journal.ppat.1003321 23853576 PMC3701714

[17] Sedelnikova OA, Pilch DR, Redon C, Bonner WM (2003) Histone H2AX in DNA damage and repair. Cancer Biol Ther 2: 233–235. 12878854 10.4161/cbt.2.3.373

[18] Moody CA, Laimins LA (2009) Human papillomaviruses activate the ATM DNA damage pathway for viral genome amplification upon differentiation. PLoS Pathog 5: e1000605 10.1371/journal.ppat.1000605 19798429 PMC2745661

[19] Park JW, Shin MK, Pitot HC, Lambert PF (2013) High incidence of HPV-associated head and neck cancers in FA deficient mice is associated with E7's induction of DNA damage through its inactivation of pocket proteins. PLoS One 8: e75056 10.1371/journal.pone.0075056 24086435 PMC3781031

[20] Mehta K, Gunasekharan V, Satsuka A, Laimins LA (2015) Human papillomaviruses activate and recruit SMC1 cohesin proteins for the differentiation-dependent life cycle through association with CTCF insulators. PLoS Pathog 11: e1004763 10.1371/journal.ppat.1004763 25875106 PMC4395367

[21] Matsuoka S, Ballif BA, Smogorzewska A, McDonald ER 3rd, Hurov KE, et al (2007) ATM and ATR substrate analysis reveals extensive protein networks responsive to DNA damage. Science 316: 1160–1166. 10.1126/science.1140321 17525332

[22] Kitagawa R, Bakkenist CJ, McKinnon PJ, Kastan MB (2004) Phosphorylation of SMC1 is a critical downstream event in the ATM-NBS1-BRCA1 pathway. Genes Dev 18: 1423–1438. 10.1101/gad.1200304 15175241 PMC423193

[23] Abraham RT (2001) Cell cycle checkpoint signaling through the ATM and ATR kinases. Genes Dev 15: 2177–2196. 10.1101/gad.914401 11544175

[24] Stucki M, Clapperton JA, Mohammad D, Yaffe MB, Smerdon SJ, et al (2005) MDC1 directly binds phosphorylated histone H2AX to regulate cellular responses to DNA double-strand breaks. Cell 123: 1213–1226. 10.1016/j.cell.2005.09.038 16377563

[25] Limoli CL, Giedzinski E, Bonner WM, Cleaver JE (2002) UV-induced replication arrest in the xeroderma pigmentosum variant leads to DNA double-strand breaks, gamma -H2AX formation, and Mre11 relocalization. Proc Natl Acad Sci U S A 99: 233–238. 10.1073/pnas.231611798 11756691 PMC117544

[26] Bassing CH, Alt FW (2004) H2AX may function as an anchor to hold broken chromosomal DNA ends in close proximity. Cell Cycle 3: 149–153. 10.4161/cc.3.2.689 14712078

[27] Celeste A, Fernandez-Capetillo O, Kruhlak MJ, Pilch DR, Staudt DW, et al (2003) Histone H2AX phosphorylation is dispensable for the initial recognition of DNA breaks. Nat Cell Biol 5: 675–679. 10.1038/ncb1004 12792649

[28] Celeste A, Difilippantonio S, Difilippantonio MJ, Fernandez-Capetillo O, Pilch DR, et al (2003) H2AX haploinsufficiency modifies genomic stability and tumor susceptibility. Cell 114: 371–383. 12914701 10.1016/s0092-8674(03)00567-1 PMC4737479

[29] Bassing CH, Suh H, Ferguson DO, Chua KF, Manis J, et al (2003) Histone H2AX: a dosage-dependent suppressor of oncogenic translocations and tumors. Cell 114: 359–370. 12914700 10.1016/s0092-8674(03)00566-x

[30] Turinetto V, Giachino C (2015) Multiple facets of histone variant H2AX: a DNA double-strand-break marker with several biological functions. Nucleic Acids Res 43: 2489–2498. 10.1093/nar/gkv061 25712102 PMC4357700

[31] Pilch DR, Sedelnikova OA, Redon C, Celeste A, Nussenzweig A, et al (2003) Characteristics of gamma-H2AX foci at DNA double-strand breaks sites. Biochem Cell Biol 81: 123–129. 10.1139/o03-042 12897845

[32] Rogakou EP, Pilch DR, Orr AH, Ivanova VS, Bonner WM (1998) DNA double-stranded breaks induce histone H2AX phosphorylation on serine 139. J Biol Chem 273: 5858–5868. 9488723 10.1074/jbc.273.10.5858

[33] Park JW, Nickel KP, Torres AD, Lee D, Lambert PF, et al (2014) Human papillomavirus type 16 E7 oncoprotein causes a delay in repair of DNA damage. Radiother Oncol 113: 337–344. 10.1016/j.radonc.2014.08.026 25216575 PMC4268372

